# Developing an optimised method for accurate wear testing of dental materials using the ‘Rub&Roll’ device

**DOI:** 10.1038/s41598-024-68873-y

**Published:** 2024-08-02

**Authors:** Eva Maier, Jan Ruben, William M. Palin, Ewald Bronkhorst, Manuel Olmos, Ragai Edward Matta, Bas Loomans

**Affiliations:** 1 Department of Operative Dentistry and Periodontology, University Hospital Erlangen, Friedrich–Alexander Universität Erlangen–Nürnberg, Glueckstrasse 11, 91054 Erlangen, Germany; 2 Department of Dentistry, Radboud University Medical Center, Nijmegen, The Netherlands; 3 Dental and Biomaterials Science, School of Dentistry, College of Medical and Dental Sciences, University of Birmingham, Birmingham, UK; 4 Department of Oral and Cranio-Maxillofacial Surgery, University Hospital Erlangen, Friedrich–Alexander Universität Erlangen–Nürnberg, Erlangen, Germany; 5 Department of Prosthodontics, University Hospital Erlangen, Friedrich–Alexander Universität Erlangen–Nürnberg, Erlangen, Germany

**Keywords:** Dental materials, Wear, Erosion, Abrasion, Laboratory test method, Resin-based composites, Dentine, Enamel, Experimental models of disease, Preclinical research, Biomedical materials, Characterization and analytical techniques, Scanning electron microscopy

## Abstract

Dental materials are challenged by wear processes in the oral environment and should be evaluated in laboratory tests prior to clinical use. Many laboratory wear-testing devices are high-cost investments and not available for cross-centre comparisons. The ‘Rub&Roll’ wear machine enables controlled application of force, chemical and mechanical loading, but the initial design was not able to test against rigid antagonist materials. The current study aimed to probe the sensitivity of a new ‘Rub&Roll’ set-up by evaluating the effect of force and test solution parameters (deionized water; water + abrasive medium; acid + abrasive medium) on the wear behaviour of direct and indirect dental resin-based composites (RBCs) compared with human molars against 3D-printed rod antagonists. Molars exhibited greater height loss than RBCs in all test groups, with the largest differences recorded with acidic solutions. Direct RBCs showed significantly greater wear than indirect RBCs in the groups containing abrasive media. The acidic + abrasive medium did not result in increased wear of RBC materials. The developed method using the ‘Rub&Roll’ wear machine in the current investigation has provided a sensitive wear test method to allow initial screening of resin-based composite materials compared with extracted human molars under the influence of different mechanical and erosive challenges.

## Introduction

Dental resin-based composites (RBCs) have been researched and developed for over 60 years, and their advancement in terms of relevant key material properties have alleviated problems of clinical wear for most restorations. However, significant concerns of sub-optimal wear characteristics still exist for modern RBC materials used for restoration of large occlusal surfaces in patient treated with severe tooth wear^1^ or for patients with parafunctional habits^2–5^. A review study on the longevity of direct resin composite restorations cited annual failure rates ranging between 0.08% and 4.9% in the posterior region with the main reason for failure being secondary caries and fracture^6^. Another recent systematic review on prospective clinical trials of direct class II or class I restorations in permanent teeth reported a mean overall survival rate of 85–90% after 10 years, where bulk fractures and wear were found responsible for about 70% of the restoration replacements^7^. Especially when rehabilitating severely worn dentitions, the restorative materials will have to face the same erosive and abrasive challenges that damaged the tooth substrates in the first place.

The significant developments and clinical success of RBC materials have likely led to more frequent selection by dental practitioners of these materials over more traditional, and mostly less conservative options, such as ceramic or metal-ceramic prostheses. Alvanforoush et al.^8^ compared the clinical success of direct RBC restorations in vital posterior teeth over two consecutive decades (1995–2005 and 2006–2016). Remarkably, the failure reason ‘wear’ decreased from 9.4 to 1.43% for each decade, whereas the occurrence of fractures increased from 28.8 to 39.1%, respectively^8^. It is also important to note that wear is often not considered as a failure, and that fractures require intervention (repair or replacement)^9^. However, wear processes can significantly weaken RBC materials, which then are more likely to fracture^6^. Therefore, continued development, refinement and standardisation of in vitro and clinical wear testing remains an important goal for researchers, manufacturers, and dentists alike.

Laboratory wear testing methods are useful for reasonably fast analysis and comparison of material types. In these aspects, they are advantageous over clinical studies that are likely to require years of data collection and are reliant upon successful patient recall. However, the design and development of accurate and reliable laboratory wear testing methods is extremely challenging since precise control of experimental variables is required^10^.

One important aspect of developing standard tests involves simplicity, low cost and reproducibility whilst maintaining as much clinical relevance as possible. Many previous and existing systems are high-cost investments or unique devices that have been developed in-house and are not readily available for cross-centre comparison, e.g. the artificial mouth concept designed by the Minnesota Dental Research Center for Biomaterials and Biomechanics^11^, the Oregon Health Science University wear tester^12^, the Regensburg simulator^13^, the BIOMAT simulator^14^ or the “Dento-Munch Robo-Simulator”^15^.

Whilst these machines may provide useful insight into wear processes, some with excellent reproduction of masticatory load patterns and clinical significance, it is impossible to compare across different test centres which is required to develop standard tests and to quickly determine the minimum material requirements for clinical use. Furthermore, the wide variability in testing parameters like load, rotation speed or mode of friction was emphasised by a recent systematic review, which reported 30–70% variability for the same material and method^16^. This strongly demonstrates a severe lack of standardisation, which is likely to contribute to lack of translational research enabling correlation between laboratory and clinical wear data.

Consequently, there is a need for a simplified in vitro wear device that allows discrimination between relevant material types and conditions. The ‘Rub&Roll’ device was developed by Ruben and Roeters^17^ aiming to fulfil these requirements. It allows wear and loading studies, that may involve additional erosive and/or abrasive challenges. The clinical relevance was demonstrated on the example of mimicking erosive wear (‘cuppings’) on the occlusal surface of natural teeth^18^.

The current study aimed to investigate the capability and sensitivity of the ‘Rub&Roll’ device by evaluating the effect of force and test solution parameters on the wear behaviour of direct and indirect resin-based composites (RBCs) compared with human molars.

## Results

### Comparison of wear behaviour of natural teeth and resin-based composites

**Figure 1:**
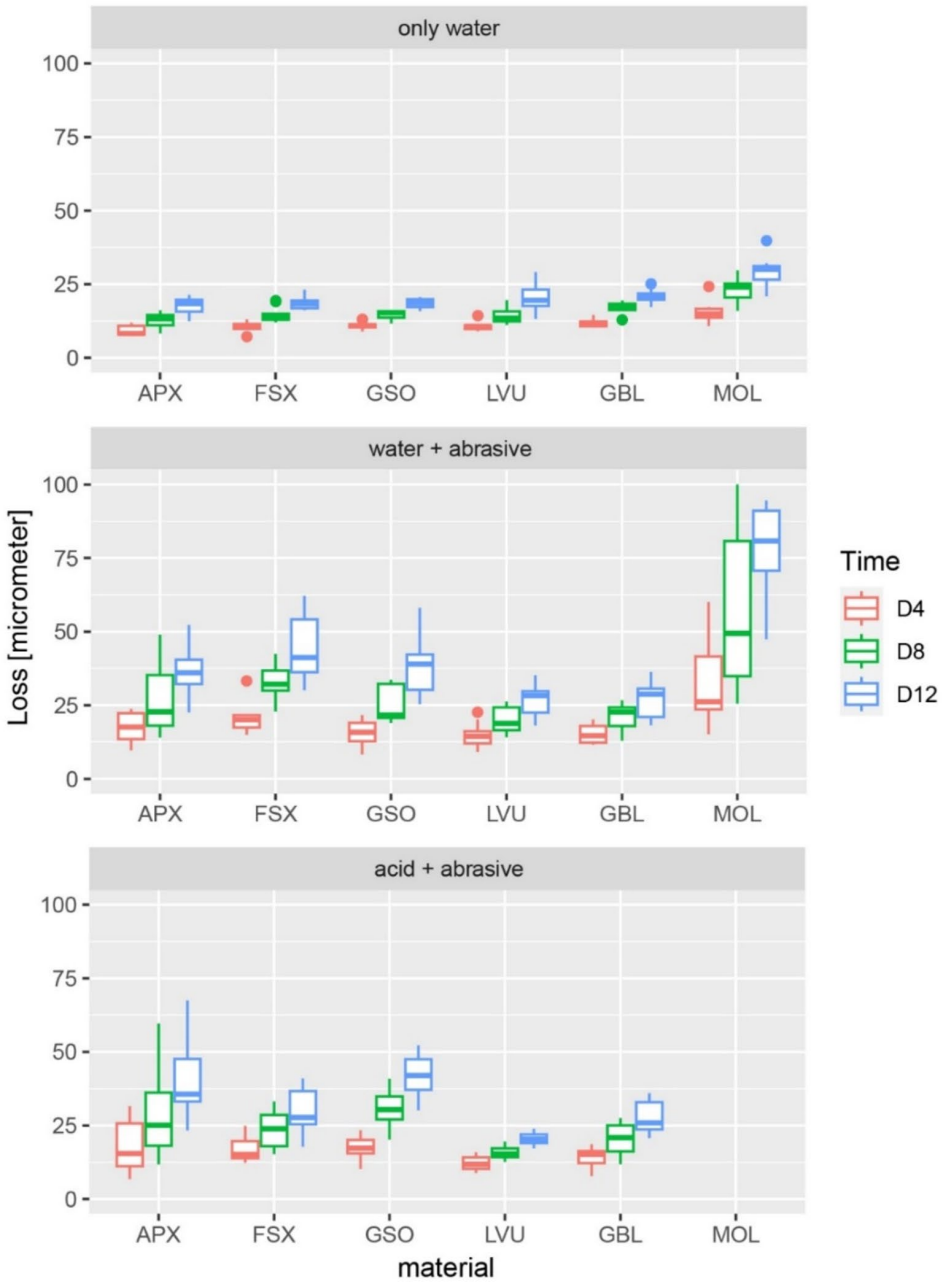
Distribution of wear by group, time, and material: interquartile range displayed from 1st to 3rd quartile, the line indicating the median; whiskers are presenting values within 1.5 times of the interquartile range, dots depict outliers outside of this range; Y-axis is limited to 100 µm, which hides molars in the acid + abrasive group. (values MOL in the acid-abrasive group [mean value ± standard deviation] = D4: 602 ± 122 µm; D8: 1428 ± 230 µm; D12: 2196 ± 326 µm); Materials: direct RBCs (APX = Clearfil APX, Kuraray; FSX = Filtek Supreme XTE, 3 M; GSO = GrandioSO, VOCO) indirect RBCs (LVU = Lava Ultimate, 3 M; GBL = GrandioBlocs, VOCO) and human molars (MOL). Mean height loss [µm] displayed after 4 (D4), 8 (D8) and 12 (D12) days.

Regardless of immersion conditions, human molars (MOL) exhibited the highest rate of wear and showed significantly more height loss than RBCs in all test groups (p < 0.001, Fig. 1).

**Figure 2:**
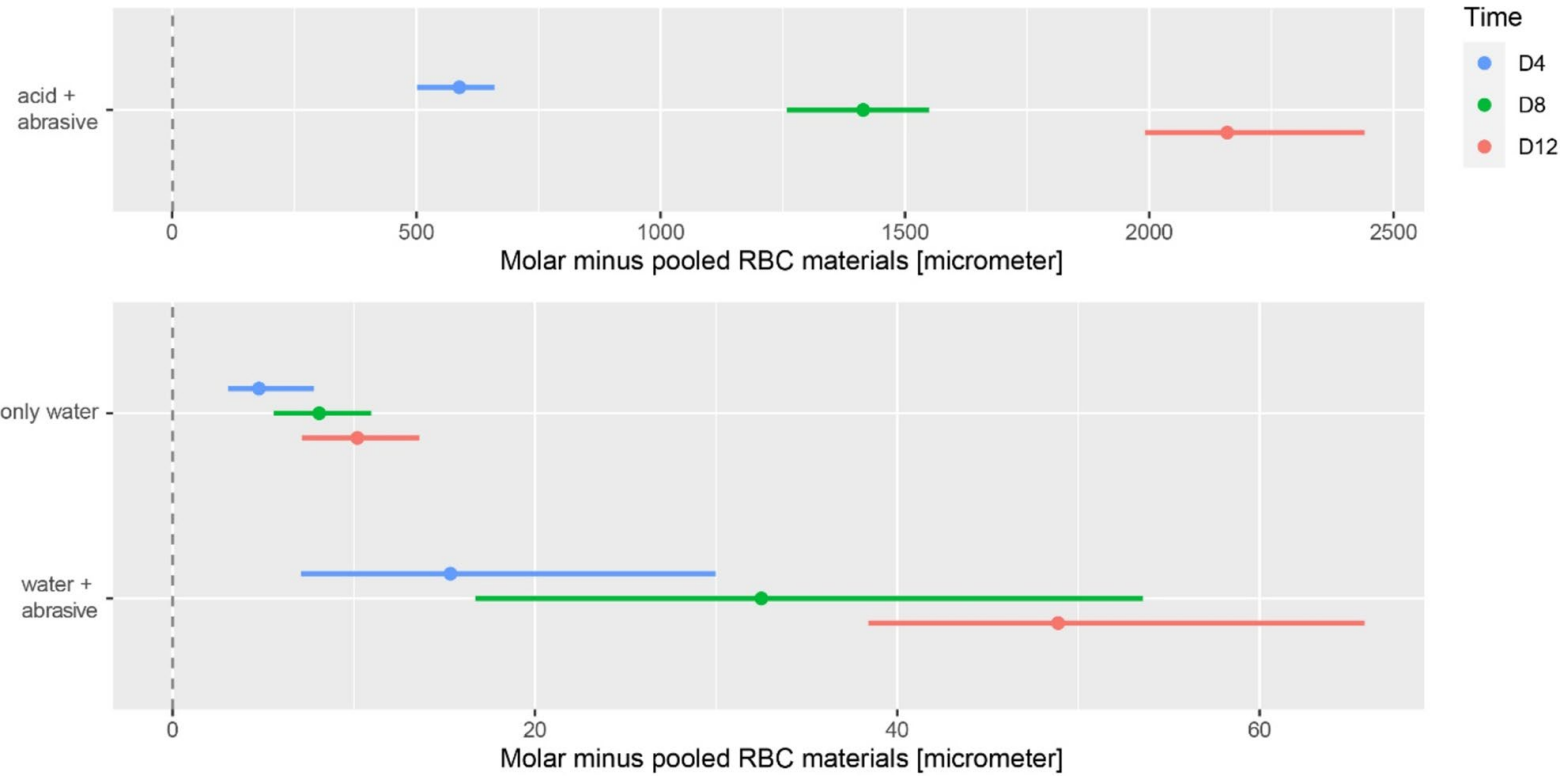
Difference in wear between human molars and pooled RBC materials [µm]; estimate of differences (dots) and 95% confidence intervals (lines) after 4 (D4), 8 (D8) and 12 (D12) days.

The wear rates of human molars increased more than that of RBCs following the addition of the abrasive to water compared with water alone at all timepoints (mean differences to pooled RBC values: 4 days: 15 µm, 95% CI 7–30 µm; 8 days: 33 µm, 95% CI 17–54 µm; 12 days: 49 µm, 95% CI 38–66 µm; in all cases p < 0.0001, Fig. 2). A substantial increase in mean height loss of human molars was observed in the lowest pH-conditions (mean differences to pooled RBC values: 4 days: 588 µm, 95% CI 501–660 µm; 8 days: 1414 µm, 95% CI 1258–1549 µm; 12 days: 2160 µm, 95% CI 1991–2440 µm).

### Recorded pH-values of different test-solutions

The significantly reduced wear resistance of human molars compared with RBC materials was further corroborated by the substantial increase in acidity of the testing solutions from group 1–3. The mean pH-values in group 1 (de-ionized water) were 6.35 ± 0.17 before and 6.82 ± 0.50 after testing. pH-values in group 2 (de-ionized water + abrasive) decreased from 6.08 ± 0.04 before testing to 5.25 ± 0.36 after testing. Increased pH-values from 3.67 ± 0.02 before testing to 4.24 ± 0.08 after testing were recorded in group 3 (acid + abrasive).

### Differences in wear behaviour between direct and indirect resin-based composite materials

**Figure 3:**
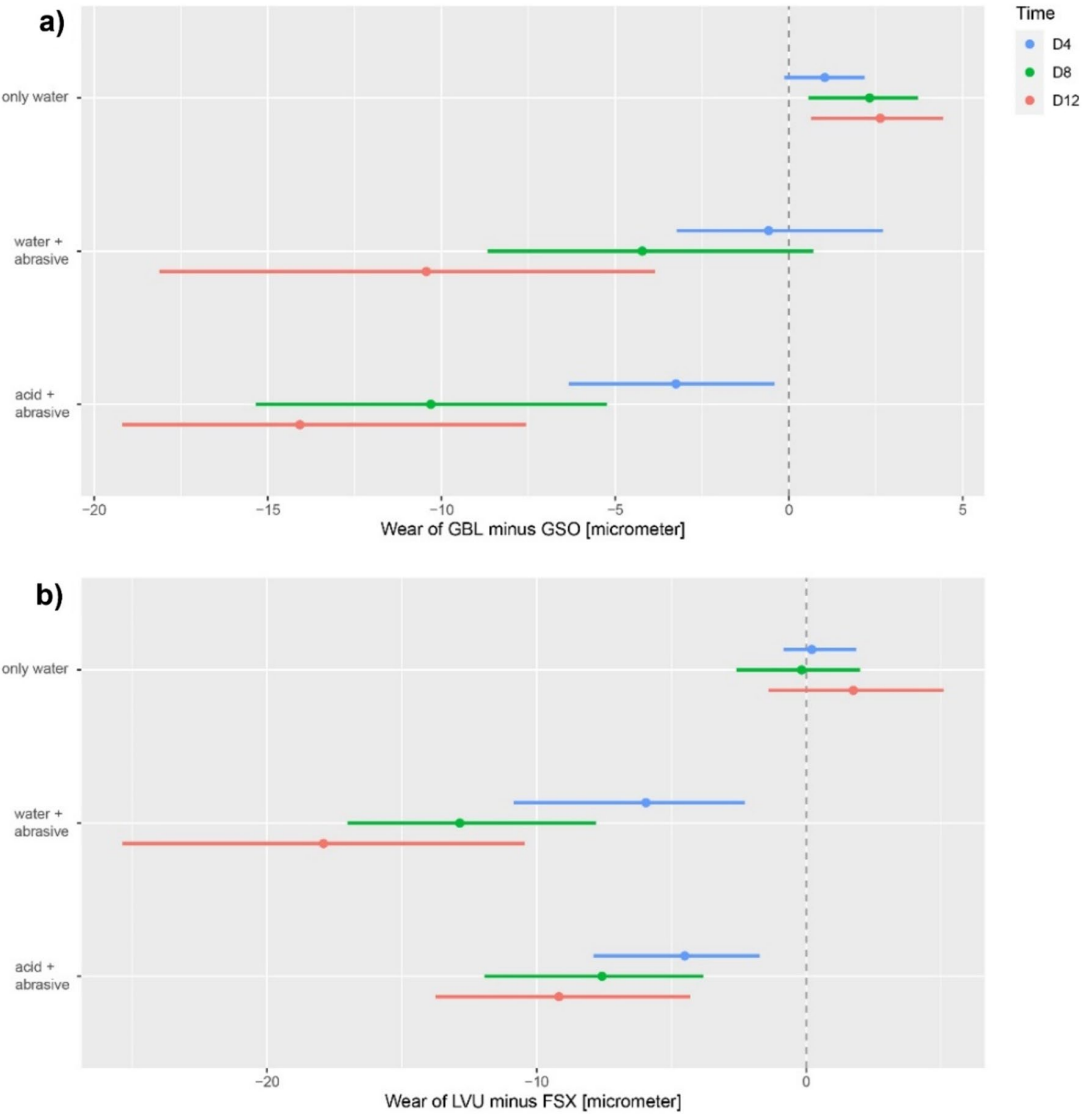
Differences in wear between indirect and direct material pairs of the same manufacturer ((**a**): GrandioBlocs [GBL] vs. GrandioSO [GSO], VOCO; (**b**): Lava Ultimate [LVU] vs. Filtek Supreme XTE [FSX], 3 M; [µm]); estimate of differences (dots) and 95% confidence intervals (lines) after 4 (D4), 8 (D8) and 12 (D12) days.

In group 1 (de-ionized water), significant differences were recorded between the direct and indirect RBCs at all time points, with the largest differences observed after 12 days (mean differences: − 2.5 µm 95% CI − 4.3 to 0.6 µm). However, under more aggressive conditions, larger differences in the wear of RBC materials were recorded dependent upon material type. For both group 2 (water + abrasive; mean differences direct minus indirect: 4 days: 2.9 µm, 95% CI 0.7–5.5 µm; 8 days: 7.6 µm, 95% CI 4.7–11.3 µm; 12 days: 12.6 µm, 95% CI 7.8–16.9 µm) and group 3 (water + acid; mean differences direct minus indirect: 4 days: 4.1 µm, 95% CI 2.0–13.7 µm; 8 days: 9.4 µm, 95% CI 5.3–13.7 µm; 12 days: 13.3 µm, 95% CI 7.6–17.5 µm), indirect RBCs exhibited significantly lower material loss compared with respective direct RBC materials (p < 0.05). These differences were shown between direct and indirect materials of the same manufacturer (Fig. 3a: GBL vs. GSO; Fig. 3b: LVU vs. FSX), which are known to have similar composition only varying in processing conditions^19^. Adding an acidic solution to the abrasive medium (group 3 vs. 2) did not result in significantly increased wear of RBC materials (p > 0.05 for all three time points).

### Average forces measured in the load simulation

The average forces evaluated in the load simulation in the universal testing machine were 21.25 ± 3.5 N for 0.5 mm protrusion, 38.81 ± 7.78 N for 1.0 mm protrusion and 48.71 ± 8.72 N for 1.5 mm protrusion.

### Qualitative microstructural analysis of the wear facets using scanning electron microscopy

**Figure 4:**
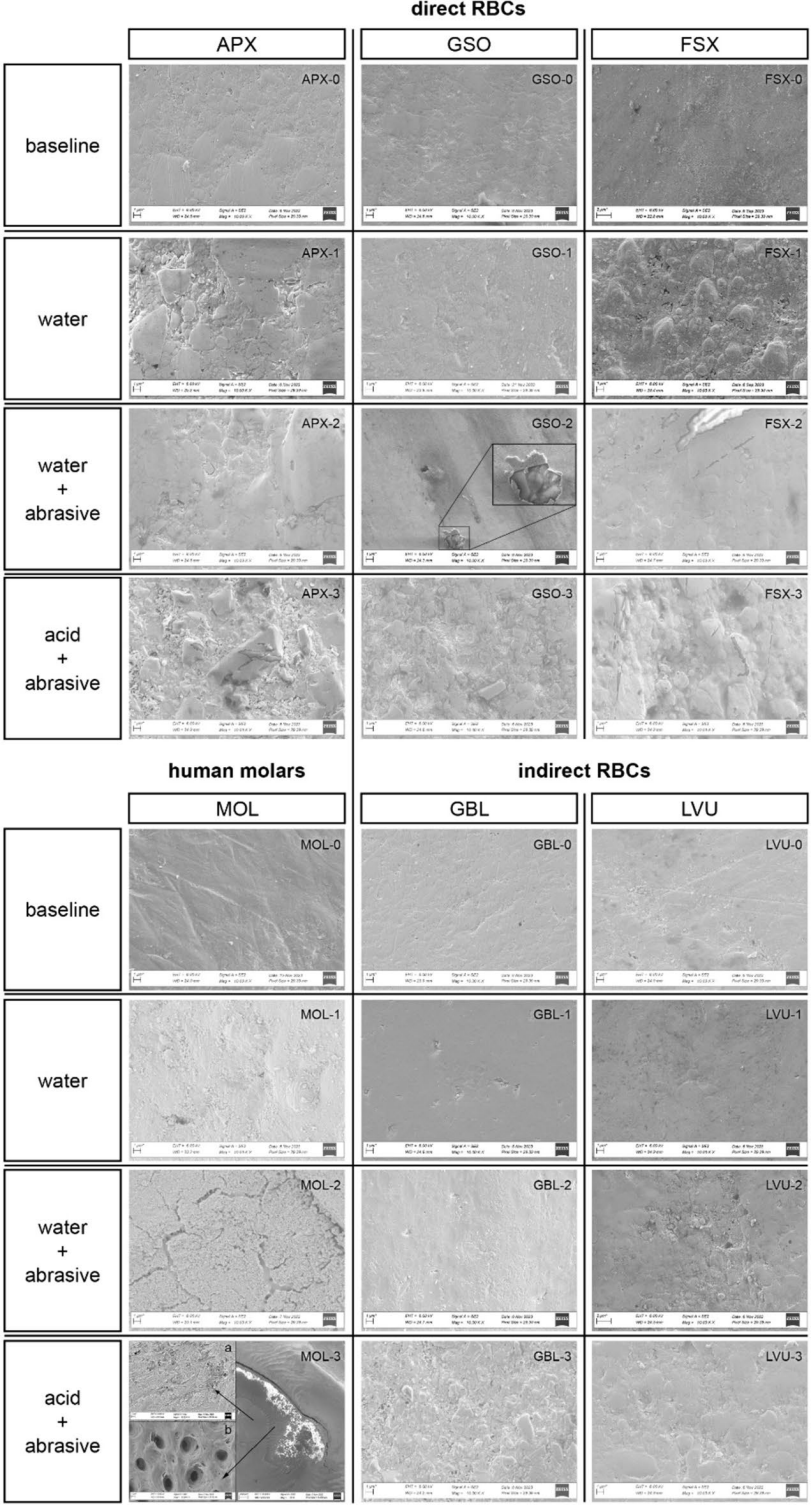
Scanning electron microscope images (6 kV, Sigma 300/ZEISS, ×10.000 [overview Mol-3 ×50]) of the pre-test (baseline) and worn surfaces of each respective group (1: only water, 2: water + abrasive, 3: acid + abrasive) for all materials tested: direct RBCs (APX = Clearfil AP-X, Kuraray; GSO = GrandioSO, VOCO; FSX = Filtek Supreme XTE, 3 M) indirect RBCs (GBL = GrandioBlocs, VOCO; LVU = Lava Ultimate, 3 M) and human molars (MOL).

Qualitative microstructural analysis of the baseline specimens and the worn surfaces after twelve days was performed for different materials in different groups (Fig. 4). At baseline, all RBC materials have a similar appearance (APX-0, GSO-0, FSX-0, GBL-0, LVU-0). In the ‘only water’ group 1 and the ‘acid + abrasive’ group 3, filler particles are more exposed, meaning more of the resin matrix was removed, in the direct materials than in their indirect counterparts worn under same condition (GSO-1 vs. GBL-1; GSO-3 vs. GBL-3; FSX-1 vs. LVU-1; FSX-3 vs. LVU-3). In the ‘water + abrasive’ group-2, a smoother surface presented for all RBC materials compared with the group with acid involved. In the respective GSO specimen, a small hole in this even surface of abraded debris reveals the underlying material surface with fillers embedded in the resin matrix system (detail GSO-2). Human molars showed only slight surface changes compared to baseline when worn in ‘only water’ (MOL-0 vs. MOL-1). When abrasive medium was added microcracks could be detected on the surface (MOL-2). Tooth specimens in the acid-abrasive solution were worn/eroded so extensively that the prismatic enamel structure was exposed (MOL-3a) and in even deeper wear areas dentinal tubules (MOL-3b) were displayed.

## Discussion

Laboratory wear testing methods aim to mimic the complex processes that teeth and dental materials are subject to within the oral environment, thereby attempting to predict clinical behaviour and long-term functionality in form of translational research. From a plethora of available devices and protocols in the field of laboratory wear testing, the ‘Rub&Roll’ wear machine aims to combine test sensitivity and flexibility concerning test parameters with achievable costs and reproducibility. The test sensitivity was supported by the current results, as there were differences monitored not only between natural tooth material and resin-based composite materials (RBCs), but as well between direct and indirect materials. Furthermore, the influence of added abrasive medium during testing on wear behaviour of teeth or tooth-shaped dental materials was demonstrated. The inclusion of flexible inserts around test specimens allowed better representation of the stress field associated with the masticatory cycle, i.e. the load absorption characteristics of the periodontal ligament. Thus, load is dissipated more effectively, which can be beneficial compared with other in vitro wear machines that do not consider this and only use stiff supports with low compliance^19^.

Regardless of the immersion conditions, human molars developed the largest material height loss. Sound human molar crowns are covered by a protective enamel layer that provides high wear resistance due to its hardness and refined prismatic structure^20,21^. The underlying softer dentin enables flexibility of natural teeth, to buffer high forces applied on the surface^18,22^. Resin-based composites on the other hand consist mainly of filler particles embedded in a resin matrix and usually exhibit similar elastic modulus to dentine, and not enamel^23^. Wear mechanisms primarily attack the resin matrix by dissolving or abrading this softer phase of the composite^24^. Such differential wear patterns, especially with larger filler particles result in a rough surface^16^.

In the current study, human enamel showed less wear resistance than all the RBC materials. The findings on tooth wear were similar to previous studies where differences were observed depending on the laboratory wear testing method applied, i.e. a higher enamel wear in direct contact and a lower wear resistance when similar abrasive media were involved^19^. In the acidic environment, the differences in wear between enamel and RBC materials were much higher. This can be explained regarding the erosive potential of enamel and dentine, which is far greater than that of RBC materials (Fig. 1). The rate of enamel dissolution significantly increases at a pH of 5.2–5.5 or lower, that of dentine already from approximately 6.7 downwards^25^. Even well polymerized RBC materials degrade primarily by water intrusion, which can be accelerated in low pH environments^26^. The mean pH-values before (3.67 ± 0.02) and after (4.24 ± 0.08) testing of the citric acid solution were therefore likely to soften the tooth substrate, accelerating wear processes on the human molars. This is supported by the fact that the mean height-loss was higher than the protrusion of the specimen at each time point. To mimic clinical processes causing severe tooth wear as a combined process of erosive and mechanical wear, it would be necessary to increase the pH-value to a less aggressive value or to combine phases of attack (acidic solution) with phases of recovery (neutral solution). The mean pH values in groups 1 and 3 increased over the testing period, which might be caused by the dissolution of calcium from the teeth. De-ionized water in group 1 lacks ionic bonds and the acidic solution in group 3 can dissolve calcium. The increase in acidity in group 2 might be caused by the degradation of natural organic components like lipids and proteins^27^.

In the two groups with abrasive medium involved, indirect RBCs exhibited significantly lower material loss compared with their respective direct RBC counterparts. For direct and indirect materials of the same manufacturer comparable material compositions (size and amount of filler particles) were provided (Table 1), and also confirmed in the current microstructural analysis and most visible when materials were tested in the ‘acid + abrasive’ group 3 (Fig. 4, GSO-3 vs. GBL-3, FSX-3 vs. LVU-3). However, the differences between chairside and laboratory-processed materials (subtractive milling from pre-polymerized blocks compared with additive and incrementally placed materials) significantly influence polymerization characteristics and mechanical properties^5,28,29^. Optimized indirect polymerization processes that use extended exposure under floodlamp (oven) curing with controlled high temperature and vacuum is likely to result in increased crosslink density and degree of conversion compared with direct handheld curing processes under ambient conditions. Consequently, this could lead to higher resistance to mechanical and chemical attack and was further confirmed by SEM where filler particles were more prominent and exposed in the direct materials, whereas smoother and more evenly embedded in their indirect counterparts (Fig. 4: GSO-3 vs. GBL-3, FSX-3 vs. LVU-3).

**Table 1:** Information revealed by the manufacturers on the composition of materials under investigation.

Material	Manufacturer	Manufacturers’ description	Composition
Clearfil AP-X (APX)	Kuraray (Tokyo, Japan)	Light-cured, radio-opaque restorative composite resin	Matrix: BIS-GMA, TEGDMA Fillers: 71 vol% inorganic fillers (0.02–17 µm): Silinated barium glass fillers Silinated colloidal silica Silanated silica fillers
GrandioSO (GSO)	VOCO (Cuxhaven, Germany)	Universal nano-hybrid packable direct resin-based composite	Matrix: BIS-GMA, TEGDMA, BIS-EMA Fillers: 89 wt%/73vol% inorganic fillers Glass ceramic fillers (average particle size 1 µm) Functionalized silica dioxide nano particles (size 20–40 nm)
Filtek Supreme XTE (FSX)	3 M (Saint Paul, Minnesota, USA)	Universal nano-hybrid packable direct resin-based composite	Matrix: BIS-GMA, UDMA, TEGDMA, BIS-EMA, PEGDMA Fillers: 78.5 wt%/63.3 vol% Silica fillers (20 nm) Zirconia fillers (4–11 nm) Zirconia–silica-cluster (0.6–20 µm)
GrandioBlocs (GBL)	VOCO (Cuxhaven, Germany)	Nanoceramic hybrid CAD-CAM bloc	86 wt% fillers Glass ceramic fillers (average particle size 1 µm) Functionalized silica dioxide nano particles (size 20–40 nm)
Lava Ultimate (LVU)	3 M (Saint Paul, Minnesota, USA)	Nano-particulate pre-polymerized indirect resin-based composite (CAD-CAM)	80 wt%, 65 vol% fillers Silica fillers (20 nm) Zirconia fillers (4–11 nm) Zirconia–silica-cluster (0.6–20 µm)

In laboratory wear testing, the choice of antagonist opposing the material under investigation influences the magnitude and character of resulting wear defects^30^. Hard antagonist materials (zirconia, stainless steel, etc.) will exhibit increased wear resistance with less change in surface characteristics thereby delivering more constant abrasive behaviour of the test material^19,31^. The current project attempted to better mimic the clinical situation of patients who received full-mouth rehabilitation with RBC materials. Therefore, a 3D-printed unfilled resin material was chosen as an antagonist, to evaluate the general option of inserting rigid rods compared to the flexible PVC-rods used in the original set-up of the ‘Rub&Roll’ wear machine in a cost-effective way. The opportunity of easily adapting and replacing the antagonist rods can be rated as a key advantage of the ‘Rub&Roll’ wear machine. Simple fine-tuning of material type and modulus, e.g. increasing filler load in the printed resin antagonist rods, could enable investigating the effect of antagonist stiffness on wear characteristics of opposing tooth tissue or restorative material. Furthermore, as transparent unfilled 3D-printed resins are commonly used for occlusal splints to protect natural dentition^32^, the new set-up of the device could be used to test the wear behaviour of different rod-materials against teeth or tooth-shaped restorations. The ‘Rub&Roll’ wear machine can therefore be adapted to current and emerging technologies in dentistry, to cost-effectively test complex scenarios of clinical wear processes.

Clinically, quantitative height-loss of teeth and restorations over time can be measured by digital impressions and direct superimposition^33^ or by taking analogue impressions and investigating the resulting gypsum or acrylic casts^34,35^. Comparing these clinical results to the current study would be necessary to translationally estimate the predictive ability of the ‘Rub&Roll’ device for clinical outcomes. Here the often-discussed question remains if clinical wear will ever be accurately replicated in laboratory wear testing in comparison across different sites^3,16^. Currently, there exist no in vitro models of wear that can exactly mimic the complex oral environment or exactly replicate the clinical consequences of abrasion and attrition, due to the multi-factorial process of wear. There have been commendable examples that attempt to mimic the clinical scenario^11,12^, however, they are usually complex, expensive to develop, and provide limited possibility for standardisation or interlaboratory comparisons. Nevertheless, the development of the method using the ‘Rub&Roll’ wear machine in the current investigation has provided a sensitive and affordable test method to allow initial screening of dental materials, presenting low acquisition costs for the device combined with the opportunity of testing multiple specimens at the same time. A comparison to clinical data is necessary, to evaluate translational relevance of this ‘Rub&Roll’ set-up.

## Methods

The extracted teeth used in the current study were obtained from the University of Birmingham’s Biobank. The collection after informed consent of the patients and the use of this waste oral tissue harvested during surgery was approved and covered by the ethical approval reference 19/SW/0198.

For the current study, the original ‘Rub&Roll’ wear machine design^17^ was adapted to enable twice the number of specimens (32 compared with 16) against a maximum of four antagonist rods. The improved design was also orientated along a vertical axis to ensure contact of the specimens with the abrasive media even when sedimented at the bottom of the testing cylinder.

Two indirect RBCs (GrandioBlocs/GBL; VOCO, Cuxhaven, Germany and Lava Ultimate/LVU; 3 M, St. Paul, Minnesota, USA), three direct RBCs (GrandioSO/GSO; VOCO, Filtek Supreme XTE/FSX; 3 M, Clearfil APX/APX; Kuraray, Tokyo, Japan), and sound upper human molars (MOL) were challenged in different groups (n = 10; Table 1). For preparation of the indirect specimens, an intraoral scan was randomly chosen from one indirectly restored patient from the Radboud Tooth Wear Project^36^. The first upper left molar was digitally extracted from the scan (inLab CAD software, Dentsply Sirona, Charlotte, North Carolina, USA) and closed at the lower surface including an indentation to create a retentive volumetric body. Specimens were milled from the indirect resin-based composite blocks (inLab MC XL milling machine, Dentsply Sirona).

For production of the direct RBCs, impressions of the indirect (milled) specimens were taken using high-precision impression silicone (Panasil Binetics, Kettenbach, Eschenburg, Germany/Miratray Mini, Hager Werken, Duisburg, Germany). Moulds were subsequently filled in increments of 2 mm and adjusted using a composite adaptation instrument (flat plastic). Increments were polymerized for the manufacturers’ recommended curing time using an LED-curing light (Bluephase 20i or G4, with an irradiance between 1050–1090 mW/cm^2^, measured using a Bluephase Meter II; Ivoclar, Schaan, Liechtenstein). A mark in the centre of the lower surface of the test piece was created to provide a macro-retentive pattern for the consecutive embedding.

Occlusal surfaces of both indirect and direct RBC specimens were polished dry at 8000 rpm using a handpiece and polishing discs (TwistDIA, Kuraray) and a wool buffing wheel to remove any polishing residue.

The RBC specimens were embedded in polymethyl methacrylate (PMMA; Autoplast, Candulor AG, Glattpark; Switzerland) to enable mounting in the ‘Rub&Roll’ wear machine. To level the cusps, RBC specimens were inverted and temporarily fixed on glass plates using heated thermoplastic impression compound (50 °C; Kerr, Herzogenrath, Germany). The PMMA powder and liquid monomer were mixed, filled into silicone moulds, and the glass plates (with inverted fixed specimens) were inserted and weighed down until set. The final embedded specimen dimensions were adjusted to 10.5 mm thickness, 15.0 mm width, 24.2 mm height using a precision drill press (HBM BF 25 Milling machine, HBM Machines, Gouda, The Netherlands). On each side of the RBC specimens, flat reference areas were added to simplify the superimposition of two consecutive profilometer scans for quantitative wear analysis.

**Figure 5:**
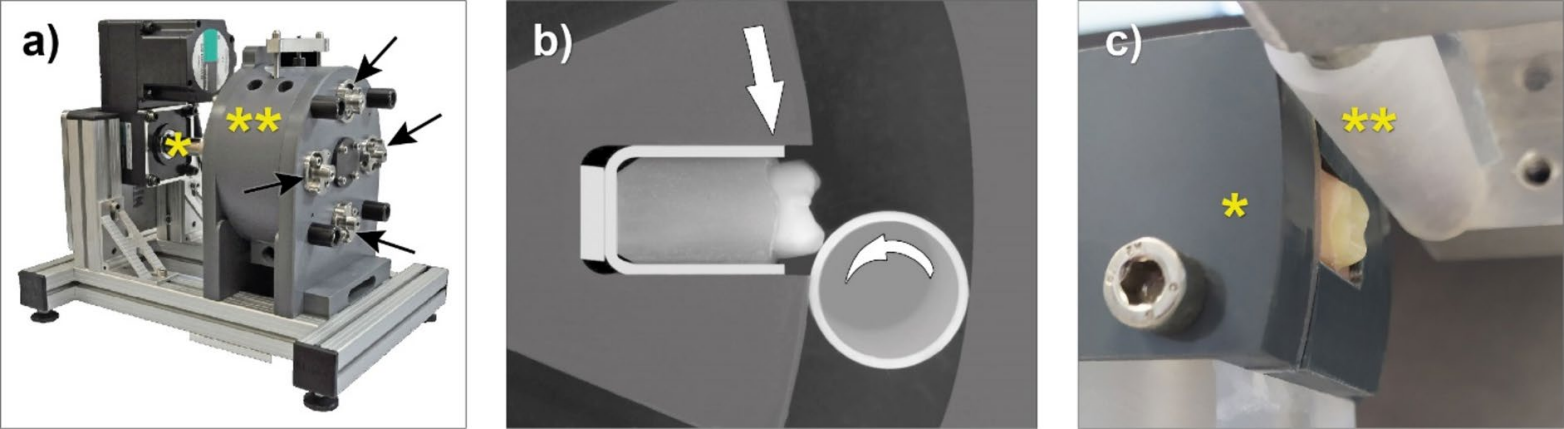
(**a**) ‘Rub&Roll’ wear machine: engine with connecting shaft driving the specimen wheel rotation (one yellow asterisk*), specimen chamber containing the specimen wheel with abrasive/erosive solution and antagonist rods (two yellow asterisks**), four fixation screws indicating positions of the antagonist rods inside the specimen chamber (black arrows); (**b**) schematic presentation of the mounted specimen with rubber inserts (1 mm on sides, 3 mm + respective protrusion on the bottom). White arrows indicating the turning direction of the antagonist rods and the specimen wheel during testing; (**c**) set-up in universal testing machine to simulate the load occurring in the ‘Rub&Roll’ wear machine: trimmed part of the ‘Rub&Roll’ specimen wheel holding one sample (one yellow asterisk*), 3D-printed antagonist rod being moved up and down by the universal testing machine, measuring applied force (two yellow asterisks**).

The new set-up aimed to not only perform erosive wear testing and contact to food boluses, but also mechanical wear testing of RBCs in contact to an antagonist material. As the originally used PVC rods were too soft to produce pure mechanical wear on the materials under investigation, antagonist material rods (68 mm length, 14 mm outer/10 mm inner diameter) were printed in a commercially available stereolithography 3D printer (Form 2/Clear V4, both Formlabs). The rotation speed of the specimen wheel was 10 rpm, with a tooth contact velocity of 68 mm/s. Using four antagonist rods, 57,600 tooth contacts per 24 h were performed. Twelve days of testing (12 × 24 h) approximated to a simulation of three years of clinical challenge, as one year was described to be mimicked by about 240,000 tooth contacts^17,37^. To ensure flexibility in the system and compliance that better represented the stress field associated with the masticatory cycle, rubber inserts (1 and 3 mm thick, respectively) were positioned at the sides and beneath the specimens (Fig. 5). The extent of specimen protrusion and thereby the applied load was therefore defined by the thickness of the rubber insert beneath the specimen. The test was started with 0.5 mm protrusion for the first two days to avoid catastrophic failures (running-in phase). After 2 days, the protrusion was changed to 1.0 mm, and after eight days to 1.5 mm to allow for a stepwise increase of load. The resulting load was simulated and measured in a universal testing machine (LS1, Lloyd instruments, United Kingdom) at the same contact chewing speed of 68 mm/s.

To simulate different oral environmental challenges, abrasive or erosive solutions were added to the ‘Rub&Roll’ machine during wear testing. The following groups were tested: group 1: de-ionized water (control); group 2: de-ionized water + abrasive medium (yellow millet seed “LaPlata”, Georg Andreas GmbH, Regensburg, Germany); group 3: abrasive medium + acidic solution (0.052 mol/l (1%) citric acid, pH 3.6, according to^38^. Solutions were changed daily; pH values were measured at the beginning and end of each test-day (pH-meter, WTW InoLab pH720, Weilheim, Germany).

Wear rates were monitored using non-contact profilometry (ProScan2100; Sensor S29/10–10,000 µm, sample rate 1000 Hz, step-size 40 µm; both Scantron, Taunton, Minnesota, USA) at baseline and after 4, 8 and 12 days measuring mean height loss. Specimens were powdered (Lava Powder, 3 M) according to the initial clinical protocol used in the Radboud Tooth Wear Project. Per RBC specimen four measurements per surface (one on each respective cusp) were recorded. Due to anatomical variations of the natural teeth sometimes only three cusps reached the levelled surface initially. For comparable quantitative analysis, the smallest of the measured values was excluded in all cases where four measurements were performed. Qualitative microstructural analysis of the worn surfaces of randomly selected samples of different materials in different solutions was performed using scanning electron microscopy (SEM, 6 kV, Sigma 300; ZEISS, Oberkochen, Germany). To compare the surface at baseline with the worn surfaces after 12 days, one additional specimen per group was produced and analysed. Specimens were shortened to 3 mm height, cleaned (3 min, 2.5% NaOCl), rinsed with water, air-dried, mounted on single stubs and sputter-coated with gold prior to investigation.

Statistical analysis was performed by using *t*-tests for comparison between two groups and ANOVA with Tukey post-hoc testing for comparison of more groups (p < 0.05). For all analyses bootstrapping with 400-fold resampling was applied to find 95% confidence intervals.

## Data Availability

The data presented in this study are available on request from the corresponding author.
